# Diurnal variation of optical coherence tomography–based macular fluid in exudative age-related macular degeneration

**DOI:** 10.1186/s40942-023-00495-4

**Published:** 2023-09-25

**Authors:** Blake H. Fortes, Aaron M. Fairbanks, Aravindh A. Nirmalan, David O. Hodge, Kevin Ferenchak, Andrew J. Barkmeier

**Affiliations:** 1 Department of Ophthalmology, Mayo Clinic, 200 First Street SW, Rochester, MN 55905 USA; 2 Department of Quantitative Health Sciences, Mayo Clinic, Jacksonville, FL USA

**Keywords:** Neovascular age-related macular degeneration, Exudative age-related macular degeneration, Wet age-related macular degeneration, Optical coherence tomography, Diurnal variation, Intraretinal fluid, Subretinal fluid, Exudation, Reproducibility

## Abstract

**Background:**

Significant diurnal fluctuation of optical coherence tomography (OCT)-based macular fluid occurs in patients with several macular conditions including diabetic macular edema (DME) and cystoid macular edema due to retinal venous occlusion (RVO). OCT imaging and analysis of macular fluid status plays a central role in clinical management of exudative age-related macular degeneration (eAMD), however diurnal variation of eAMD OCT findings has not yet been formally studied. Herein, we investigate whether clinically meaningful fluctuation of OCT-based macular fluid occurs in patients with eAMD.

**Methods:**

Prospective observational study. Patients with eAMD and intra- and/or sub-retinal fluid on early AM OCT were enrolled to receive two consecutive OCT scans at least four hours later. Retinal layers were manually segmented on all OCT rasters and AM-to-PM and PM-to-PM image pairs were analyzed for total retinal and neurosensory retinal volume changes within the central 1 and 3 mm ETDRS subfields. Finally, two masked retinal specialists analyzed all OCT image pairs for qualitative differences that may impact clinical management.

**Results:**

21 patients with eAMD and fluid on OCT were recruited between January 2020 and November 2021. There was no mean difference between AM and PM central 3 mm total retinal volume (p = 0.56), central 3 mm neurosensory retinal volume (p = 0.25), central 1 mm total retinal mean thickness (p = 0.96), or central 1 mm neurosensory retinal mean thickness (p = 0.63), nor were any differences identified in PM-to-PM control comparisons. Qualitative analysis by two masked experts identified no clinically significant differences between any AM-to-PM OCT image pairs.

**Conclusions:**

No significant diurnal variation in OCT-based macular fluid or thickness was identified in patients with eAMD, either quantitatively or qualitatively.

## Background

Age-related macular degeneration (AMD) is a leading cause of vision loss in individuals over age sixty in western countries [1, 2]. In AMD, a progressive deposition of drusen develops between the outer retina and Bruch’s membrane within the macula. This leads to progressive photoreceptor cell death and loss of central vision. AMD can be divided into early, intermediate, and late stages, with the late stage being further subdivided into non-exudative (atrophic) and exudative (eAMD) forms. In eAMD, unabated macular neovascularization (MNV) leads to leakage of fluid and lipoprotein exudates into the intraretinal and subretinal spaces [1]. Rapid vision loss may occur in this setting.

Current management of eAMD centers on reducing vascular permeability and stabilizing neovascularization with intravitreal anti-vascular endothelial growth factor (anti-VEGF) pharmacotherapy, which reduces the risk of further vision loss [1]. Retina specialists compare serial optical coherence tomography (OCT) images throughout the treatment course to evaluate for potentially subtle changes in macular fluid that may prompt changes in treatment or follow-up intervals. The optimal anti-VEGF treatment interval or agent may vary between patients, between eyes of the same individual, and may change over time. Recent American Society of Retina Specialists Preferences and Trends surveys demonstrate the critical importance of OCT findings in eAMD management. Of over 1,000 retina specialists surveyed, 90% stated that OCT features are the primary factor that determines whether a patient’s treatment interval should be extended [3]. Furthermore, approximately 90% of retina specialists reported that they assess responsiveness to treatment by assessing for new or persistent macular fluid on OCT, and 75% used changes in OCT-based central retinal thickness to assess treatment response [4].

Diabetic macular edema (DME) is another condition involving vision-threatening exudation and fluid accumulation within the macula. Several studies have identified significant diurnal variation of DME on OCT, with the zenith of edema occurring in the morning and tapering to a nadir as the day progresses [5–9]. Similar diurnal variation of macular edema is also seen on OCT in patients with branch and central retinal vein occlusions (RVO) and in patients with uveitis [10, 11]. However, to our knowledge, no studies have investigated potential diurnal variation in eAMD. In comparison to DME and CME related to RVO or uveitis, relatively subtle changes in fluid on OCT may be more likely to influence treatment decisions in eAMD. Therefore, determining whether and to what extent OCT imaging in eAMD may vary throughout the day is critically important, with potentially significant implications for the timing of future appointments, OCT imaging interpretation, and eAMD management decisions.

Our prospective observational study aims to address this evidence gap by investigating the potential existence and magnitude of diurnal variation in OCT-based macular fluid in patients with eAMD through objective comparisons between early morning and afternoon imaging. Our study also includes masked comparisons of morning and afternoon OCT scans by retina specialists to evaluate for qualitative changes that may impact clinical management.

## Methods

Diurnal variation was investigated via quantitative comparison of manually segmented (performed by AMF, BHF, and AAN) morning and afternoon OCT-based retinal thickness and volume measurements, a control analysis of the repeatability of OCT manual segmentation-based quantitative assessment (performed by AMF, BHF, and AAN) was also performed. In addition, an expert qualitative assessment (by retina specialists AJB and KF) was performed on back-to-back PM OCT images acquired within minutes of each other. This prospective study received approval from the Mayo Clinic Institutional Review Board. Data were collected in accordance with HIPAA 1996 guidelines, and the study conformed to the tenets of the Declaration of Helsinki.

### Subjects

Patients with new or previously diagnosed eAMD and the presence of macular fluid on an early morning OCT (before 9:00 AM) were recruited from retina clinics at Mayo Clinic between January 2020 and November 2021. Subjects provided written informed consent prior to study enrollment. All patients received a comprehensive dilated examination with best corrected visual acuity, intraocular pressure, and OCT imaging. Each patient was diagnosed with eAMD by a retina specialist who had access to original treatment naïve OCT imaging and fluorescein angiography. In cases where exudative fluid was present in both eyes, the eye with better visual acuity was enrolled. Patients without clearly visible intra- and/or sub-retinal fluid on the early AM OCT scan and those with limited OCT image quality were excluded from the study.

### Retinal imaging and quantitative analysis

All OCT images were captured using Heidelberg Spectralis OCT running HRA2/Spectralis Family Acquisition Module 6.16.6.0 (Heidelberg Engineering GmbH, Heidelberg, Germany) Images were captured in reference scan mode with auto-alignment to ensure consistency between image acquisitions. The OCT acquisition was reviewed to ensure a signal strength/quality of ≥ 25 and a standard ART of at least 9. Back-to-back PM OCT scans were scheduled 4 + hours following the initial AM OCT acquisition and taken approximately one minute apart in follow-up mode.

Heidelberg Eye Explorer software (version 1.10.4.0; Heidelberg Engineering GmbH) was used to manually segment retinal layers (AMF, BHF, and AAN) on all horizontal OCT rasters into Neurosensory Retinal (ILM to retinal pigment epithelium [RPE]; including subretinal fluid, when present) and Total Retinal sections (Internal limiting membrane [ILM] to Bruch’s Membrane [BM]; including sub-RPE material, when present). Mean thickness/volume was recorded from these retinal sections within the central 1 and 3 mm zones of the Early Treatment of Diabetic Retinopathy Study (ETDRS) subfields.

Potential diurnal variation was assessed by comparing AM-to-PM total retinal volume and neurosensory retinal volume within the central 3 mm ETDRS subfield, and central 1 mm total retinal and neurosensory retinal mean thickness. Analyses were repeated on back-to-back PM scans to assess test-retest repeatability of image acquisition and manual segmentation.

### Qualitative analysis

**Fig. 1:**
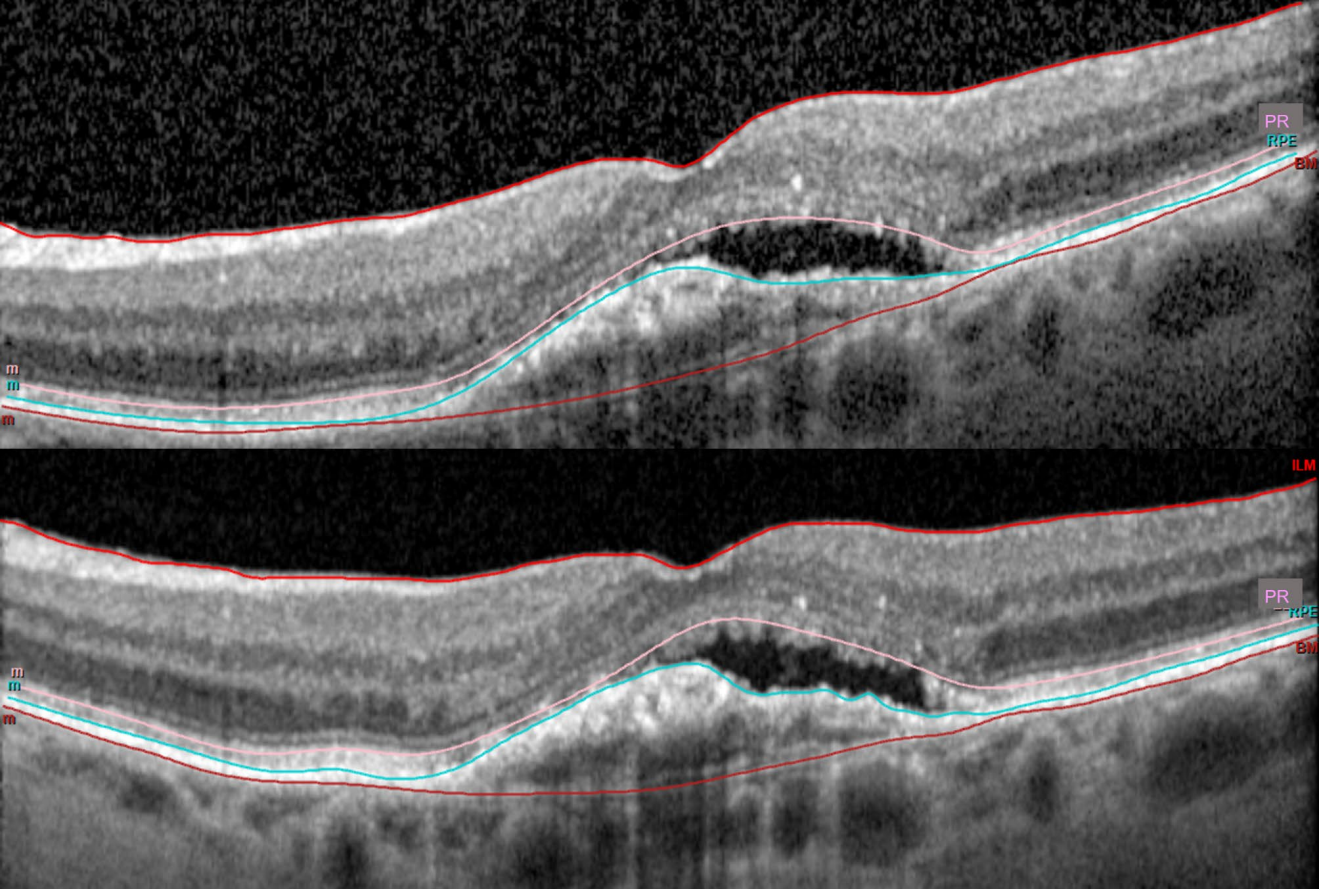
Morning (top) and afternoon (bottom) manually segmented optical coherence tomography (OCT) scans from a single patient that demonstrates a subtle difference in the amount of subretinal fluid as determined by masked graders. This difference was classified as subtle and would not impact clinical management

Two retina specialists (AJB, and KF) qualitatively graded all AM-to-PM and PM-to-PM OCT raster image sets while masked to both image timing and quantitative analysis to assess for potential differences in exudative fluid that may impact clinical management. De-identification was performed by assigning each patient a randomized study ID known only to the investigator conducting the test (BHF). Pairs of OCT raster sets were graded using the following scale: (1) no change, allowing subtle inter-raster differences but with neither raster set worse overall; (2) mild, subclinical difference in raster sets that would not alter clinical management; or (3) clear difference in fluid between raster sets that may alter management. Figure 1 is an example of a manually-segmented corresponding raster AM/PM image pair with a subtle subretinal fluid difference that would not alter clinical management (grade = 2). Inter-grader differences were adjudicated by a secondary masked review to reach consensus.

### Statistical analysis

Statistical analysis was performed using SAS Software version 9.4 (SAS Institute, Inc., Cary, NC, USA). Retinal thickness/volume measurements from morning and afternoon scans were transferred to an Excel spreadsheet (Microsoft Corp., Redmond, WA) and compared using a paired t-test. The retinal thickness and volume measurements of consecutive afternoon scans were prepared for calculation of coefficients of repeatability using methods described by Bland and Altman to assess for reproducibility of scans and manual segmentation [12]. Statistical significance was defined as an alpha level for statistical significance of p < 0.05.

## Results

21 patients with eAMD and intra- or subretinal fluid on OCT were recruited from January 2020 to November 2021. Mean age at enrollment was 81 years and all patients self-identified as Caucasian, although other racial and ethnic groups were not excluded. Of the 21 patients, five were male and 16 were female. Two patients were newly diagnosed with eAMD and thus anti-VEGF treatment naïve. Mean duration of anti-VEGF pharmacotherapy prior to study participation was 41 months. Bevacizumab was the most frequent prior anti-VEGF agent in this cohort, with 18 patients most recently receiving bevacizumab and the remaining three receiving aflibercept at the prior visit (Table 1).

**Table 1:** Baseline demographics and clinical characteristics of patients with exudative age-related macular degeneration

Characteristics	n=21 (%)
Age at enrollment (years) Mean (median, range)	81 (80, 69–96)
Sex	
Male	5 (24)
Female	16 (76)
Race	
Caucasian	21 (100)
Anti-VEGF naïve	
Yes	2 (10)
No	19 (90)
Duration of eAMD diagnosis (months) Mean (median, range)	41 (23, 0-135)
Most recent anti-VEGF agent	
Bevacizumab	18 (86)
Aflibercept	3 (14)

Abbreviations: anti-VEGF: anti-vascular endothelial growth factor, eAMD: exudative age-related macular degeneration

**Fig. 2:**
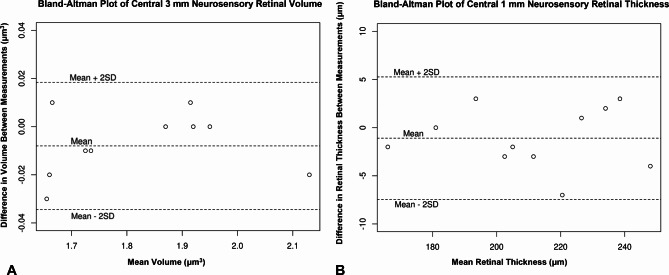
Bland-Altman plots for central 3 mm neurosensory retinal volume **(A)** and central 1 mm neurosensory retinal thickness **(B)** measurements between consecutive afternoon scans that demonstrate the reproducibility of consecutive OCT scans and manual segmentation

There was no mean difference between AM and PM central 3 mm total retinal volume (AM: 2.69 µm^3^ vs. PM: 2.68 µm^3^; p = 0.56), central 3 mm neurosensory retinal volume (AM: 1.96 µm^3^ vs. PM: 1.95 µm^3^; p = 0.31), central 1 mm total retinal mean thickness (AM: 403 μm vs. PM: 403 μm; p = 0.96), or central 1 mm neurosensory retinal mean thickness (AM: 253 μm vs. PM: 251 μm; p = 0.77) [Table 2]. Control analyses comparing immediate consecutive PM images similarly identified no differences in any of these measures (central 3 mm total retinal volume: PM1: 2.40 µm^3^ vs. PM2: 2.39 µm^3^; p = 0.54; central 3 mm neurosensory retinal volume: PM1: 1.80 µm^3^ vs. PM2: 1.81 µm^3^; p = 0.07; central 1 mm total retinal mean thickness: PM1: 337 μm vs. PM2: 335 μm; p = 0.58; central 1 mm neurosensory retinal mean thickness PM1: 211 μm vs. PM2: 212 μm; p = 0.28) [Table 3]. Bland-Altman plots of central 3 mm neurosensory retinal volume and central 1 mm neurosensory retinal thickness measurements between consecutive PM scans were performed to demonstrate reproducibility of consecutive OCT scans and manual segmentation (Fig. 2A and B).

**Table 2:** Comparison of AM versus PM Total and Neurosensory Retinal Volume and Central Subfield Mean Thickness

Parameters	AM (N = 21)	PM (N = 21)	*p*
Central 3 mm neurosensory retinal volume (ILM to RPE) [µm^3^]	1.96	1.95	0.25
Central 1 mm neurosensory retinal mean thickness (ILM to RPE) [µm]	253	251	0.63
Central 3 mm total retinal volume (ILM to BM) [µm^3^]	2.69	2.68	0.56
Central 1 mm total retinal mean thickness (ILM to BM) [µm]	403	403	0.96

Abbreviations: ILM: internal limiting membrane, RPE: retinal pigment epithelium, BM: Bruch’s membrane

**Table 3:** Comparison of Total and Neurosensory Retinal Volume and Central Subfield Mean Thickness on Consecutive PM OCT Imaging

Parameters	PM1 (N = 11)	PM2 (N = 11)	*p*
Central 3 mm neurosensory retinal volume (ILM to RPE) [µm^3^]	1.80	1.81	0.07
Central 1 mm neurosensory retinal mean thickness (ILM to RPE) [µm]	211	212	0.28
Central 3 mm total retinal volume (ILM to BM) [µm^3^]	2.40	2.39	0.54
Central 1 mm total retinal mean thickness (ILM to BM) [µm]	337	335	0.58

Abbreviations: ILM: internal limiting membrane, RPE: retinal pigment epithelium, BM: Bruch’s membrane

Qualitative analysis by two masked retinal specialists identified no clinically significant differences between any masked AM-to-PM OCT comparisons. 8 of 21 (38%) subjects had subtle subclinical AM-to-PM imaging changes (4 worse AM, 4 worse PM). No differences were identified in any PM-to-PM qualitative comparisons. Among the eight AM-to-PM masked subjective image comparisons in which subtle subclinical changes were noted, quantitative thickness differences aligned with the direction of qualitative changes in 7/8 (88%) subjects.

## Discussion

This study identified no significant diurnal variation of macular fluid or thickness in patients with eAMD and macular fluid on OCT. Several analyses were performed including quantitative OCT analyses of the central neurosensory retina and subretinal fluid (ILM to RPE), as well as the full-thickness retina and sub-RPE space (ILM to Bruch’s membrane) (Tables 2 and 3). Manual OCT raster segmentation was performed to ensure that retinal layers were appropriately delineated and retinal thickness and volume measurements were accurate. Furthermore, no clinically significant differences were identified between morning and afternoon OCT images in qualitative analyses performed by retina specialists masked to both image timing and quantitative measurements.

These results contrast with prior studies that identified diurnal variation of OCT-based macular fluid in DME, RVO, and uveitis, all of which demonstrated a peak in macular fluid early in the morning and a subsequent decrease in fluid later in the day [5–9]. One study by Kotsidis et al. reported improvement of visual acuity in addition to decreases in central subfield mean thickness (CST) and total macular volume (TMV) from the morning to the afternoon in patients with DME [7]. Another observational case series demonstrated a consistent decrease in macular thickness over the course of the day in four of 10 subjects with DME [6]. The waking value, obtained at 8 AM, was higher than any other value taken later in the day. However, in contrast to the study by Kotsidis et al., no consistent change in visual acuity was observed.

It remains unclear why clinically relevant diurnal variation was not present in our prospective cohort of patients with eAMD. Potential explanations may relate to sources of fluid (retinal versus choroidal vasculature), known differences in the magnitude of vascular leakage, and differences in RPE pump function (potentially decreased in AMD), all of which likely impact the balance and rate of fluid accumulation and clearance. Differences in pathophysiology, local environment, cytokine levels, and potentially a differential impact of systemic blood pressure, dependent positioning, and fluid balance may also affect diurnal fluctuation in retinal fluid on OCT in these conditions. In addition, most patients were already undergoing anti-VEGF treatment, which would have impacted the local environment and VEGF levels. Patients with eAMD-related macular fluid while receiving anti-VEGF pharmacotherapy, however, are likely the most clinically relevant eAMD cohort because new treatment-naïve eAMD is typically managed similarly regardless of the magnitude of fluid.

Quantitative OCT image analysis is more technically challenging in patients with eAMD in comparison to conditions that have predominant retinal vascular leakage and relatively minimal outer retinal, RPE, and sub-RPE pathology. The heterogeneity of pathology between Bruch’s membrane and retinal photoreceptors makes segmentation more challenging, but advances in SD-OCT image resolution and segmentation capabilities have increasingly enabled work in this area. Hanumunthadu et al. investigated OCT-based retinal thickness repeatability in eAMD and found that a change of greater than 31 μm in Spectralis OCT-derived retinal thickness measurement in the central macular subfield was necessary to detect a true clinical change [13]. Strengths of our study include its prospective design, inclusion of actively treated patients with eAMD which increases this study’s clinical relevance, manual segmentation that demonstrated excellent reproducibility on consecutive afternoon OCT scans and was not subject to automated segmentation errors that could potentially impact retinal thickness and volume measurements, and de-identification of scans when presented to expert graders who were masked to image acquisition times and quantitative analysis.

Limitations of this study include the modest sample size and the fact that serial OCT scans were not obtained throughout the day, or much earlier in the day, as they were in some of the studies discussed above. However, we designed the study to ensure clinically relevant timing with initial scans as early in the AM as possible (maximum thickness in non-AMD studies), and to identify potential changes occurring during normal clinic hours. A significantly larger sample size may have been able to detect a statistically significant mean quantitative change in OCT parameters, but it is unlikely that statistical differences would have been clinically significant. This study could have utilized multiple serial OCTs or required a longer interval between imaging, but we scheduled imaging four or more hours later for afternoon scans because patients who presented for a morning retina clinic examination were occasionally scheduled for a same-day intravitreal injection in the early afternoon in our practice, and enrollment in this protocol minimized patient inconvenience.

## Conclusions

We report this initial investigation of whether diurnal variation of OCT-based exudative fluid occurs in patients with eAMD. We identified no significant diurnal variation in exudation in patients with eAMD, either quantitatively or qualitatively. Mild qualitative changes were equally likely to improve or worsen later in the day. These findings are reassuring for clinicians and clinical investigators that the diurnal timing of OCT imaging for patients with eAMD is unlikely to significantly impact either clinical management or clinical trials.

## Data Availability

The datasets during and/or analysed during the current study available from the corresponding author on reasonable request.

## Meeting Presentation

This research was presented at the Retina Society Annual Meeting 2022 on November 3rd, 2022 in Pasadena, CA and the Macula Society Annual Meeting on February 17th, 2023 in Miami, FL.

## Abbreviations

AMD: Age-related macular degeneration
Anti-VEGF: Anti-vascular endothelial growth factor
CME: Cystoid macular edema
DME: Diabetic macular edema
eAMD: Exudative age-related macular degeneration
OCT: Optical coherence tomography
RVO: Retinal vein occlusion
